# Facile synthesis of SrCO_3_ nanostructures in methanol/water solution without additives

**DOI:** 10.1186/1556-276X-7-305

**Published:** 2012-06-15

**Authors:** Lishuo Li, Rongyi Lin, Zhangfa Tong, Qingge Feng

**Affiliations:** 1 School of Chemistry and Chemical Engineering, Guangxi University, Nanning, Guangxi 530004, People’s Republic of China; 2 National Engineering Laboratory for Hydrometallurgical Cleaner Production Technology, Institute of Process Engineering, Chinese Academy of Sciences, Beijing 100190, People’s Republic of China

**Keywords:** Nanoparticle, Crystallization, SrCO_3_, Refluxing

## Abstract

Highly dispersive strontium carbonate (SrCO_3_) nanostructures with uniform dumbbell, ellipsoid, and rod-like morphologies were synthesized in methanol solution without any additives. These SrCO_3_ were characterized by X-ray diffraction, field emission scanning electron microscopy, and N_2_ adsorption-desorption. The results showed that the reaction temperature and the methanol/water ratio had important effects on the morphologies of SrCO_3_ particles. The dumbbell-like SrCO_3_ exhibited a Broader-Emmett-Teller surface area of 14.9 m^2^ g^−1^ and an average pore size of about 32 nm with narrow pore size distribution. The formation mechanism of the SrCO_3_ crystal was preliminary presented.

## Background

Recently, nanomaterials with different morphologies have attracted great attention for their promising applications such as optical materials, efficient catalysts, drug-delivery carriers [1-3], etc. Strontium carbonate (SrCO_3_) is one of the important reagents used in firework, pigment, and electron manufacturing [4]. There are two main usages of SrCO_3_: they are used in the production of cathode ray tubes and ferrite magnets for small direct-current motors [5]. However, SrCO_3_ with different morphologies may own different potential usages. For example, SrCO_3_ with a needle-like crystal is used in optical polymers to reduce birefringent phenomena [6]. A sphere-shaped crystal with a diameter less than 1 μm is favorable for high-temperature electric components. So far, SrCO_3_ with various morphologies such as hierarchical branches, hexagonal prisms, straw-like, pancake, ellipsoid, needle, flower ribbon, bundle, dumbbell, sphere, and rod-like have been reported [5,7-13].

Various methods have been reported on the preparation of SrCO_3_ nanostructures including hydrothermal [14], microwave-assisted [9], microemusion-mediated solvothermal methods [5], etc. Although nanoscale SrCO_3_ with special morphologies was obtained, the preparation processes were complex and strict, such as high-pressure, high-temperature, and tedious synthetic procedures as well as high cost were required. Aside from that, large-scale synthesis of SrCO_3_ nanostructures still remains a considerable challenge.

In this work, a new facile way was reported to synthesize SrCO_3_ nanostructures by continuously carbonizing Sr(OH)_2_ with CO_2_ in methanol/water solution without additives. The effects of the reaction temperature and the methanol/water (m/w) molar ratio on the morphology evolution were investigated. This method is simple, low-cost, and easy to control in producing large-scale monodisperse SrCO_3_ nanostructures.

## Methods

**Figure 1:**
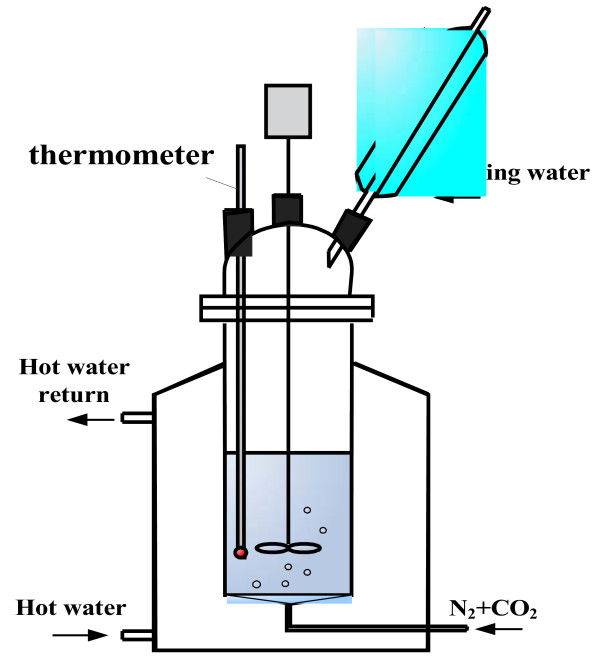
Schematic drawing of the reactor.

Sr(OH)_2_·8H_2_O was purchased from Alfa Aesar (Ward Hill, MA, USA). Other reagents were purchased from Beijing Reagents Co., Ltd. (Beijing, China) All reagents used in our experiments were of analytical grade. Experiments were carried out in a 150-ml reactor with a refluxing system. The reaction temperature was controlled using a thermostatic bath, as shown in Figure 1.

In the typical experiment, several grams of Sr(OH)_2_·8H_2_O were dissolved into the methanol/water solution and kept at room temperature for 24 h. The concentration of the solution was kept at 0.05 mol l^−1^ for all the experiments. The solution was put into the reactor and stirred using a propeller agitator with a speed of about 800 rpm. Mixed CO_2_ of 100 ml min^−1^(80 ml min^−1^ N_2_ + 20 ml min^−1^ CO_2_) was induced into the reactor and lasted for 30 min. Then, the gas was cut off and continuously agitated for another 2 h. Finally, the solution was naturally cooled to room temperature. The products were separated by centrifugation and washed with deionized water and ethanol alternately for three times. The obtained carbonate samples were dried at 60°C for 24 h.

The products were characterized by field emission scanning electron microscopy (FESEM; JSM-6700 F, JEOL, Akishima-shi, Japan) and X-ray diffraction (XRD; X’pert PRO MPD, PANalytical B.V., Almelo, The Netherlands); patterns of carbonate were recorded on a diffractometer (using Cu Kα radiation; *λ* = 0.154 nm) operating at 40 kV/30 mA. A scanning rate of 0.2° s^-1^ was applied to record the patterns. The N_2_ adsorption-desorption isotherms were measured at 77 K using an automated surface area and pore size analyzer (QUADRASORB SI-MP, Quantachrome Instruments, Boynton Beach, FL, USA).

## Results and discussion

**Figure 2:**
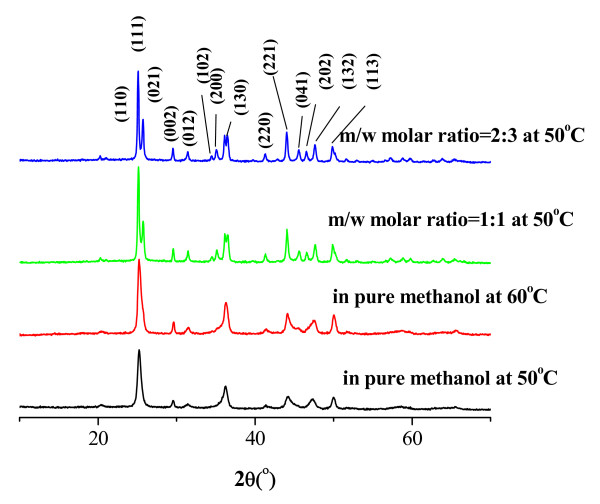
**XRD pattern of as-synthesized SrCO**_**3**_**.**

The XRD patterns in Figure 2 confirm the SrCO_3_ obtained by carbonating Sr(OH)_2_ with CO_2_ in the methanol/water system. All peaks in these patterns can be indexed as orthorhombic phase (JCPDS No. 84–0418) with lattice constants *a* = 5.107 Å, *b* = 8.414 Å, *c* = 6.029 Å; *α* =  *β* =  *γ* = 90. By comparing the XRD patterns of the as-synthesized SrCO_3_ crystal with the standard, diffraction peaks located in 2*θ* (in degrees) of 20.53, 25.35, 29.71, 31.59, 36.41, 41.42, 44.36, 47.39, and 50.10 are readily indexed as the (110), (111), (002), (012), (130), (220), (221), (132), and (113) planes for SrCO_3_, respectively. The XRD results indicate that the SrCO_3_ nanomaterials are well crystallized, and no impurity species are found.

From the XRD pattern, we can know that the SrCO_3_ from the pure methanol solution have poor crystallinity than those from the methanol/water solution (Figure 2; some crystal planes such as (021), (102), and (200) are only observed in the SrCO_3_ from the methanol/water solution. The results indicate that the SrCO_3_ from the methanol/water solution has better developed crystal plane.

**Figure 3:**
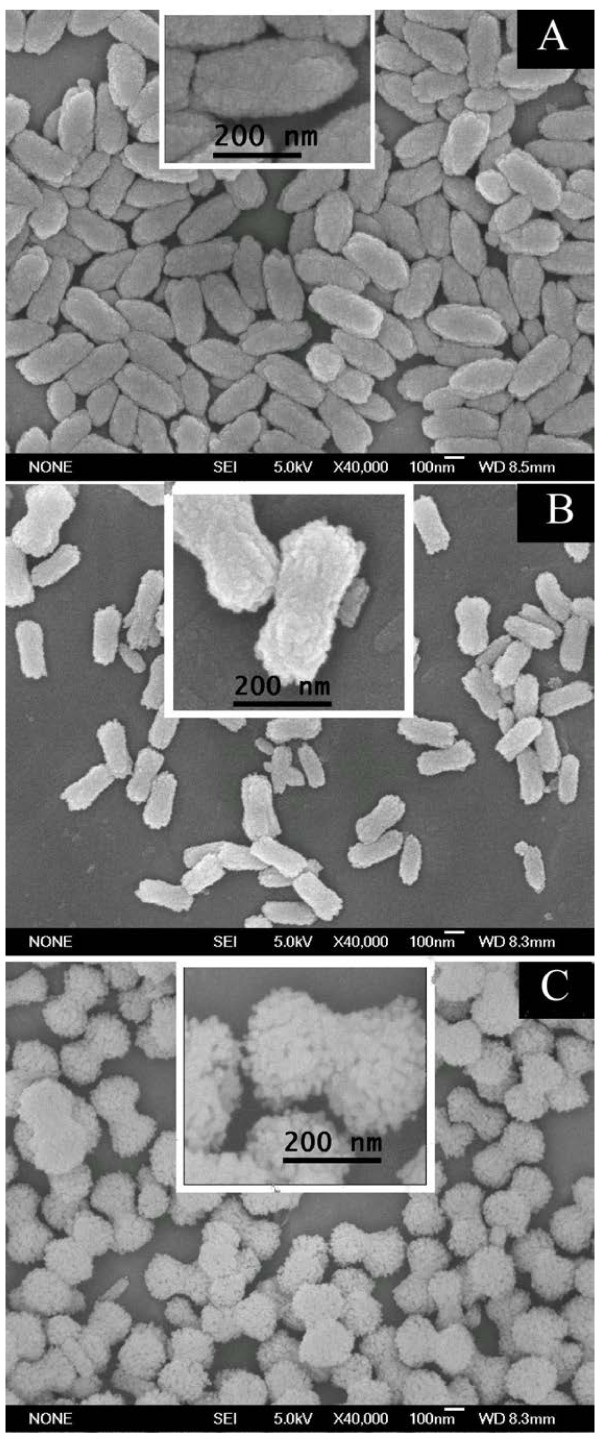
**SEM images of SrCO**_**3**_**products synthesized in pure methanol at (A) 70°C, (B) 60°C, (C) 50°C.** Insets are the magnification.

**Figure 4:**
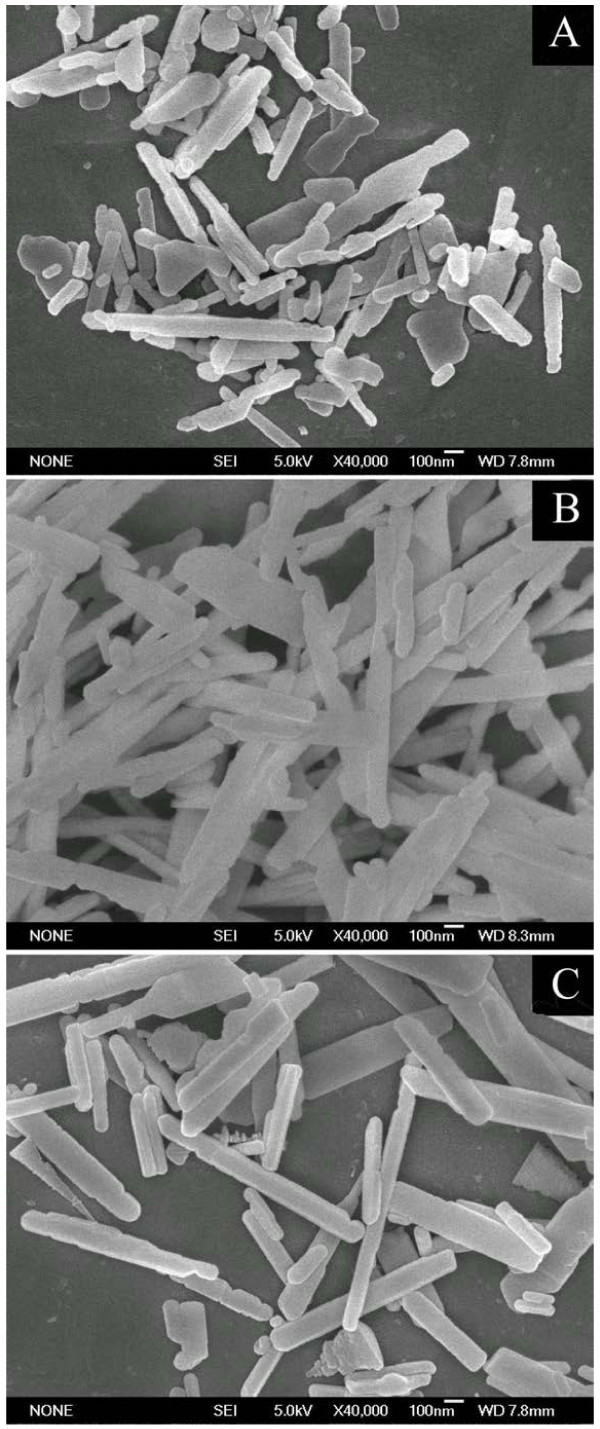
**SEM images of as-synthesized SrCO**_**3**_**particles at 50°C with different molar ratios of methanol/water.** (**A**) 3:2, (**B**) 1:1, (**C**) 2:3.

The morphologies of the products characterized by FESEM were shown in Figures 3 and 4. Figure 3 shows the morphology evolution of the products obtained at different temperatures in pure methanol. All these products have characteristics of being highly monodisperse and uniform. Ellipsoidal particles with a long axis of 350 nm and a short axis of 180 nm were obtained when a reaction temperature of 70°C was presented (Figure 3A). As the temperature was decreased to 60°C, the products were rod-like with a diameter of 110 nm and a length of about 250 nm. However, some particles have a trend to swell up, turning into dumbbell-like (Figure 3B inset). Finally, when 50°C was presented, uniform dumbbell-like particles with a handle diameter of 160 nm and a top diameter of about 200 nm were observed, and the length of the particles is about 340 nm (Figure 3C). It is obvious that these dumbbell-like particles are constructed by small nanocrystallites with a diameter of about 20 nm (Figure 3C inset). The particles seem to have a mesoporous structure, which will be confirmed by the results of the N_2_ adsorption-desorption measurement later.

In order to investigate the effect of the m/w ratio on the morphology of SrCO_3_, the crystals were prepared in m/w ratios of 3:2, 1:1, and 2:3. Figure 4 shows the morphologies of the products from the different m/w ratios. It is interesting that the rod-like SrCO_3_ with different length-diameter ratios were observed in most cases. The results indicated that the m/w ratio has a great effect on the morphology of products. When the ratio of m/w is 3:2 (Figure 4A), irregular plate-like products with a few short rods have been observed in the picture; the rods had a diameter of 90 nm and a length of 600 nm. As the m/w ratio was changed to 1:1, monodisperse rod-like products with a diameter of 120 nm and a length of 1.2 μm were observed (Figure 4B). When the m/w ratio is 2:3, the morphologies (Figure 4C) of the products were similar with those obtained in the m/w ratio of 1:1; it seems that the crystallinity of the products is better than that of Figure 4B.

**Figure 5:**
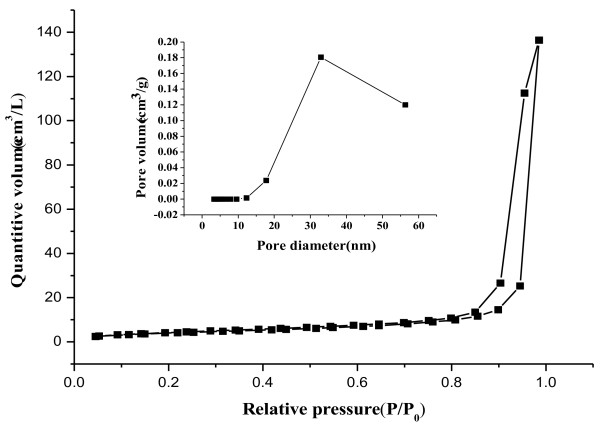
**N**_**2**_**adsorption-desorption isotherms of dumbbell-like SrCO**_**3**_**in pure methanol at 50°C.** The inset is the corresponding pore size distributions.

The high porosity properties of the dumbbell-like SrCO_3_ products were confirmed by the measurement of the Brunauer-Emmett-Teller (BET) surface area and the N_2_ adsorption-desorption isotherms (Figure 5). The BET specific surface area is 14.9 m^2^ g^−1^, which is smaller than those of the mesoporous SrCO_3 _[15] and hierarchical mesoporous SrCO_3_ submicron spheres [16]. The reason can be ascribed to the relative larger diameter of the constructing particles and the larger total pore volume of 0.18 cm^3^ g^−1^. According to IUPAC recommendations [17], the observed hysteresis of the dumbbell-like product was a characteristic of a type III isotherm with a type H3 hysteresis loop in the *P*/ *P*_0_ > 0.8. This means that the mesopores in the reign size of 12 to 32 nm were presented (Figure 5 inset). Moreover, the observed hysteresis loop near to *P*/ *P*_0_ ≈ 1 suggests that pores >50 nm were also presented [18]. This may be explained that mesopores of 12 to 32 nm ascribed to the auto-assembled stacks of uniform nanosphere, while the large pores >50 nm attributed to the aggregation of the dumbbell-like particles. It was reported that materials with a mesoporous structure possessed higher chemical reactivity due to their higher mass transportation performance [19].

Although the exact formation mechanism of these morphologies of the SrCO_3_ crystal is still not clear at present, our results show that the reaction temperature and the m/w ratio have great effects on the morphology. The m/w ratio or the polarity of the mixed solvent had a great effect on the morphology which had been reported by Lou et al. [17] and Zhang et al. [20]. Studies showed that alcohols can affect the dielectric constant of the medium and change the crystal growth rate [21]. When pure methanol was presented, the -OH of methanol might adsorb to the nuclei of the crystals and change its surface energy and then the morphology of the products. As the temperature was increased, the vibration of -OH groups in methanol was more rapid and absorption effects were weakened [22]. Thus, the morphology of the product has little change at relatively high temperatures of 60°C and 70°C (Figure 3A,B). However, when the methanol/water solution was presented, the forming hydrogen bond between methanol and water prevented the absorption to the nuclei, and rod-like products growing along the *c*-axis were obtained.

## Conclusions

Highly dispersive SrCO_3_ nanostructures with unique ellipsoid, dumbbell, and rod-like morphologies were successfully synthesized by a facile way in pure methanol or methanol/water solution without additives. The morphology of SrCO_3_ nanostructures can be controlled flexibly by adjusting the reaction temperature and the m/w ratio. N_2_ adsorption-desorption result reveals that this dumbbell-like SrCO_3_ has a mesoporous structure. It is expected that these SrCO_3_ nanostructures can be used in photocatalysis and electronic manufacturing in the future.

## Competing interests

The authors declare that they have no competing interests.

## Authors’ contributions

LL carried out the experiments studied on the crystal morphology, participated in the sequence alignment, and drafted the manuscript. Dr. RL designed the research programs and guided the experiment’s progress. Professors QF and ZT participated in the sequence alignment. All authors read and approved the final manuscript.

## Authors’ information

LL is a Ph.D. on chemical engineering science. He researches on crystal growth and design at Guangxi University. Dr. RL works as an associate researcher at the Institute of Process Engineering, Chinese Academy of Sciences. In the National Engineering Laboratory for Hydrometallurgical Cleaner Production Technology, he works on crystal growth and morphology controlling. Professors QF and ZT are experts of chemical engineering at Guangxi University.
